# Analysis of influencing factors for 5-year transfusion-related adverse events in a tertiary hospital using real-world data

**DOI:** 10.3389/fmed.2026.1748319

**Published:** 2026-02-12

**Authors:** Zhaolan Ma, Hongyan Li, Huizhen Liu, Yue Cheng, Yuling Wu

**Affiliations:** 1 Department of Blood Transfusion, Huangshan City People’s Hospital, Huangshan, China; 2 Department of Hematology, Huangshan City People’s Hospital, Huangshan, China

**Keywords:** case-control study, influencing factors, real-world data, risk assessment, transfusion adverse reactions

## Abstract

**Background:**

Transfusion-related adverse events (TrAEs), though relatively uncommon, significantly impact patient safety, with multifactorial mechanisms necessitating risk identification through real-world data.

**Objective:**

This study aimed to identify independent risk factors for TrAEs over a 5-year period in a tertiary hospital to inform clinical prevention strategies.

**Methods:**

A retrospective case-control study was conducted from January 2020 to December 2024, including 84 patients with documented TrAEs and 336 matched controls without reactions. Data on demographics, transfusion history, blood product attributes, vital signs, laboratory parameters, and outcomes were extracted from hospital records. Multivariate logistic regression was used to identify independent risk factors.

**Results:**

The observation group exhibited significantly higher values in age, number of prior transfusions, and prevalence of hypertension, diabetes mellitus, cardiovascular disease, and malignancy compared to the control group (*P* < 0.05). Patients in the internal medicine department had a higher risk of TARs than those in other departments (*P* < 0.05). Regarding transfusion characteristics, the transfusion volume in the observation group was significantly higher than that in the control group (*P* < 0.05). At 1-h post-transfusion, body temperature, heart rate, respiratory rate, systolic blood pressure, white blood cell count, and C-reactive protein in the observation group were significantly higher, while hemoglobin levels were lower than those in the control group (*P* < 0.05). In terms of clinical management, the observation group showed significantly higher rates of antipyretic-analgesic use, corticosteroid use, ICU admission, and longer hospital stays compared to the control group (*P* < 0.05). Multivariate logistic regression analysis identified increasing age, higher number of prior transfusions, comorbidities of hypertension, cardiovascular disease, malignancy, and larger transfusion volume as independent risk factors for TARs (*P* < 0.05).

**Conclusion:**

TrAEs are influenced by patient age, transfusion history, comorbidities, and transfusion volume. Enhanced monitoring and tailored transfusion strategies are recommended for high-risk patients to improve safety.

## 1 Introduction

Transfusion adverse reactions (TARs) represent clinical complications triggered by immune or non-immune mechanisms during transfusion therapy, manifesting from mild cutaneous eruptions to fatal shock and directly compromising patient safety (1). Pathophysiologically, TARs are classified into allergic reactions, febrile non-hemolytic transfusion reactions (FNHTR), and hemolytic reactions, with allergic responses and FNHTR constituting the majority of documented cases (2). Epidemiological studies indicate substantial heterogeneity in TAR incidence worldwide, with reported rates ranging from 0.5 to 5%, attributable to variations in surveillance sensitivity, definitional criteria, and population demographics (3). Intrinsic patient factors, including advanced age, female sex, and specific comorbidities such as malignancies or autoimmune disorders, have been associated with elevated reaction risks, potentially due to immune dysregulation or metabolic alterations (4). A history of prior transfusion events is particularly significant, as repeated exposures heighten recurrence likelihood through sensitization pathways, notably in IgE-mediated allergic responses (5). Blood product attributes, such as component type (e.g., red blood cells, platelets, plasma), storage duration, and leukoreduction extent, serve as critical modulators of reaction frequency and severity (6). Real-world data (RWD), derived from routine clinical practice and integrating electronic health records, transfusion repositories, and adverse event reporting systems, offer large-scale, longitudinal epidemiological insights that address external validity gaps in conventional clinical trials (7). Recent RWD-based investigations have elucidated roles of clinical contexts (e.g., emergency versus elective transfusions), transfusion indications (e.g., surgical support versus chronic anemia management), and concomitant medications (e.g., antihistamines or immunosuppressants) in modulating TAR risks (8). Laboratory parameters, including serum tryptase, complement levels, and inflammatory markers like C-reactive protein (CRP), provide biomarker evidence for mechanistic insights, facilitating clinical subtyping and early detection (9). Collectively, extant research has initiated the development of TAR risk prediction models via multicenter cohorts and registry analyses, encompassing multidimensional factors related to patients, blood products, and healthcare settings (10).

Notwithstanding the accrued epidemiological landscape of TARs, persistent limitations impede clinical translation (11). Most evidence stems from single-center retrospective datasets characterized by restricted sample sizes and brief follow-ups, hindering capture of long-term trends, rare reaction phenotypes, or geographical disparities and thus undermining generalizability (12). RWD are inherently susceptible to information biases, including underreporting, misclassification, and missing data, especially in high-volume clinical environments with inconsistent surveillance rigor, potentially leading to substantial underestimation of true incidence (13). Methodologically, cross-sectional or retrospective designs predominate, absent prospective validation cohorts, which precludes establishment of temporal sequences and causal inferences; confounders like patient comorbidities or concurrent therapies are frequently inadequately adjusted (14). Prevailing analyses often concentrate on isolated reaction subtypes (e.g., solely allergic reactions or FNHTR), yet TARs exhibit inherent heterogeneity, necessitating integrated evaluation of multifactorial interactions—for instance, synergistic effects between blood product properties and patient immune status are commonly neglected (15). Additionally, inter-institutional discrepancies in reporting standards (e.g., divergent severity grading systems) challenge data harmonization, and investigations targeting special populations (e.g., pediatric, geriatric, or multimorbid patients) remain scarce, constraining tailored preventive strategy formulation.

To address the aforementioned limitations, this investigation employed a 5-year real-world dataset from a tertiary hospital to systematically examine the determinants of adverse transfusion reactions through a retrospective analysis. A notable strength of this study is the integration of longitudinal dynamic follow-up data (2020–2024), which encompasses a large-scale cohort and multivariable parameters, including patient baseline characteristics, transfusion history, blood product attributes, laboratory indices, and clinical management protocols. Standardized definitions for adverse reactions and severity grading were applied to minimize bias. Utilizing a case-control design coupled with multivariate statistical models, potential confounding variables were controlled to accurately identify independent risk factors, such as age, transfusion history, blood product type, and transfusion volume. The primary aims were to delineate the incidence, distribution patterns, and core influencing factors of adverse transfusion reactions within the hospital setting, thereby providing high-level empirical evidence to inform the optimization of transfusion protocols, risk stratification, and preventive measures.

## 2 Materials and methods

### 2.1 General information

Data for this study were retrieved from the information system of the transfusion department at a tertiary Grade-A hospital in China, covering an 5-year period from January 2020 to December 2024. As a regional medical center, the hospital maintains a stable annual transfusion volume and a comprehensive transfusion record system, meeting the criteria for real-world data research. A retrospective case-control design was adopted. Given the rarity of transfusion reactions and the necessity for detailed, manual extraction of clinical variables (e.g., precise transfusion history, comorbidities, pre-medications) from electronic records, a full-sample analysis of all transfusion episodes over the 5-year period was not feasible. The case-control design is an efficient approach for studying rare outcomes. The observation group comprised all documented cases of transfusion-related adverse reactions during this period, totaling 84 cases, based on the hospital’s annual adverse reaction reports. The control group consisted of transfusion patients without documented adverse reactions during the study period. Using computer-generated random numbers, controls were randomly selected from this pool at an approximate 1:4 ratio to cases, resulting in 336 included subjects. No matching on baseline characteristics was performed. The actual sample sizes of 84 cases and 336 controls exceeded this requirement, ensuring sufficient statistical power. All data were extracted from the Hospital Information System (HIS) and transfusion records, with anonymization procedures applied to protect patient privacy. To ensure data independence, the unit of analysis was the patient. For patients in the observation group, only their first documented adverse reaction event during the study period was included. For patients in the control group, if they had multiple transfusion episodes without reaction, one episode was randomly selected for data extraction. This design prevents clustering effects from patients with numerous transfusions.

### 2.2 Inclusion and exclusion criteria

Inclusion criteria: ➀ Patients who received any blood product transfusion (including red cell suspension, plasma, platelets, or cryoprecipitate) at the hospital between January 2020 to December 2024; ➁ Age ≥ 18 years to exclude pediatric patients and minimize bias due to physiological differences; ➂ Complete electronic medical records, including demographic information (e.g., age, sex), transfusion details (e.g., product type, volume), and adverse reaction reports if applicable, to ensure traceability.

Exclusion criteria: ➀ Missing or incomplete medical records, such as absence of transfusion time, product batch number, or basic demographic data; ➁ Pregnant or lactating women; ➂ Participants in other interventional clinical trials during the same period.

### 2.3 Equipment information

(1) Blood product storage equipment: Helmer Scientific blood bank refrigerators (model i.Series) were used, equipped with digital temperature monitoring systems that continuously recorded storage temperatures (2–6°C for red blood cells, below −18°C for plasma and cryoprecipitate). Temperatures were automatically logged every 4 h, with alarm systems ensuring compliance with AABB standards. Routine calibration was performed monthly by the hospital’s equipment department.

(2) Transfusion equipment: Baxter International Inc. disposable transfusion sets (model Fenwal) were employed for all transfusions. These sets included 170–260 μm filters to remove microaggregates and reduce adverse reaction risks. Nurses strictly followed manufacturer guidelines regarding flow rate (4–6 mL/min for adults) and line flushing to ensure safety.

(3) Monitoring equipment: Philips Healthcare IntelliVue MX series monitors were used for real-time vital sign tracking during transfusions. Parameters included heart rate, blood pressure, respiratory rate, and oxygen saturation, automatically recorded at 5-min intervals. Devices were regularly calibrated by clinical engineering staff to ensure accuracy and consistency.

All equipment was integrated into the hospital’s quality management system, with maintenance records regularly reviewed to exclude device-related confounding factors.

### 2.4 Research methods

(1) Study design: A retrospective case-control study was conducted using data extracted from the HIS and transfusion records over the 5-Year period to minimize recall and selection bias.

(2) Data collection process: Two independently trained researchers performed data extraction. The initial data extraction included patient identifiers, transfusion dates, product information, adverse reaction records, and clinical details. Data cleaning involved removing duplicates, validating missing values through medical record review, and entering variables into standardized forms. Variables encompassed patient demographics (age, sex), transfusion details (product type, volume, frequency), adverse reaction types, and potential confounders (comorbidities, medication history). All data were anonymized using identification codes. Data collection occurred from January to March 2025, with cross-verification and arbitration by a senior physician in cases of discrepancy; overall consistency exceeded 95%.

(3) Variable definition and standardization: Transfusion adverse reactions were defined per AABB guidelines: allergic reactions (e.g., urticaria, pruritus, dyspnea), febrile reactions (temperature increase ≥ 1°C within 4 h post-transfusion with chills), and others (e.g., hemolytic reactions, circulatory overload). Transfusion history referred to any prior transfusion record. Definitions were standardized and reviewed by the transfusion department prior to data collection.

(4) Quality control: Multiple measures were implemented, including predefined codebooks, regular research meetings to discuss anomalies, and random rechecks of 10% of samples with an error rate < 2%.

### 2.5 Observation indicators

(1) Age (years): Defined as the patient’s age at transfusion, extracted from electronic records using birth and transfusion dates. For analysis, ages were grouped as < 40 years (young), 40–60 (middle-aged), and > 60 (elderly).

(2) Sex (male/female): Biological sex as recorded in medical records; no other categories were included.

(3) Transfusion history (yes/no): Defined as any prior blood product transfusion before the current episode, verified via electronic records.

(4) Blood product type: Included red cell suspension, plasma, platelets, and cryoprecipitate, classified based on transfusion records. Products met national standards following hospital blood bank testing.

(5) Transfusion volume (units): Defined as the number of units transfused per episode, with standard units (e.g., 200 mL for red cells or plasma, 250 mL for platelets). Volumes were converted from transfusion records.

(6) Adverse reaction type: Categorized as allergic, febrile, or other (e.g., hemolytic, circulatory overload), assessed using hospital-developed report forms completed within 24 h post-transfusion. Severity was noted (e.g., mild rash vs. severe dyspnea).

(7) Medication history: Recorded as the prophylactic use of medications within 24 h prior to transfusion, specifically antihistamines (e.g., loratadine, diphenhydramine) or antipyretics (e.g., acetaminophen), extracted from electronic medication records. This variable was collected to account for potential confounding, recognizing that such pre-medication is often administered to patients perceived to be at higher risk for reactions.

### 2.6 Statistical methods

Statistical analyses were performed using IBM SPSS Statistics (version 26.0). All tests were two-tailed with significance set at *P* < 0.05. Descriptive statistics: Normally distributed continuous data (e.g., age, transfusion volume) were presented as mean ± standard deviation (verified by Shapiro–Wilk test); categorical data (e.g., sex, reaction type) as frequencies and percentages. Group comparisons: Continuous variables used independent *t*-tests; categorical variables used χ^2^ tests or Fisher’s exact test for expected frequencies < 5. Univariate analysis assessed associations between each variable and adverse reactions, calculating crude odds ratios (ORs) with 95% confidence intervals (CIs) for categorical variables and *t*-tests or Mann–Whitney U tests for continuous variables. To identify independent risk factors while adjusting for confounders, multivariate binary logistic regression was employed. The initial full model included all variables deemed clinically relevant or showing association (*P* < 0.05) in univariate comparisons (Table 1): age, sex, transfusion history, number of prior transfusions, transfusion indication (surgery/other), hypertension, diabetes mellitus, cardiovascular disease, malignancy, antihistamine use, hospital department (internal medicine/surgery/other), surgery type (emergency/elective/no surgery), and transfusion volume. Given the high correlation between transfusion history (yes/no) and number of prior transfusions (see Response to Comment 4), only the number of prior transfusions was retained as a more granular measure. A backward stepwise elimination procedure (likelihood ratio test, removal threshold *P* > 0.10) was applied to this initial set to derive a parsimonious final model. The model’s goodness-of-fit was evaluated using the Hosmer-Lemeshow test, and multicollinearity was assessed via the variance inflation factor (VIF). The variable ‘antihistamine use’ was excluded from model construction due to the high likelihood of reverse causality (prophylactic use in high-risk patients). The final model’s fit and discrimination were assessed as described below. Model fit was evaluated via Hosmer–Lemeshow test (*P* > 0.05 indicating good fit), with adjusted ORs and 95% CIs reported. Multicollinearity was assessed (variance inflation factor < 10) to ensure model stability.

**TABLE 1:** Comparison of baseline characteristics.

Variable	Observation group (*n* = 84)	Control group (*n* = 336)	Statistic	*P*-value
Age (years)	58.68 ± 15.80	54.35 ± 9.84	*t* = 3.148	0.002
Gender [male, n (%)]	48(57.14)	195 (58.04)	χ^2^ = 0.022	0.499
BMI (kg/m^2^)	24.32 ± 3.15	23.87 ± 2.79	*t* = 1.288	0.198
Transfusion history [yes, n (%)]	57(67.86)	151 (44.94)	χ^2^ = 14.120	<0.001
Number of prior transfusions	2.53 ± 1.23	1.82 ± 0.91	*t* = 5.927	< 0.001
Transfusion indication [surgery, n (%)]	44 (52.38)	130 (38.69)	χ^2^ = 5.191	0.023
Transfusion indication [trauma, n (%)]	16 (19.05)	78 (23.21)	χ^2^ = 0.671	0.413
Transfusion indication [anemia, n (%)]	16 (19.05)	76 (22.62)	χ^2^ = 0.501	0.479
Transfusion indication [other, n (%)]	8 (9.52)	52 (15.48)	χ^2^ = 1.944	0.163
Hypertension (yes, n (%))	36 (42.86)	100 (29.76)	χ^2^ = 5.263	0.022
Diabetes mellitus [yes, n (%)]	29 (34.52)	85 (25.30)	χ^2^ = 3.912	0.048
Cardiovascular disease [yes, n (%)]	26 (30.95)	65 (19.35)	χ^2^ = 5.334	0.021
Malignancy [yes, n (%)]	14 (16.67)	30 (8.93)	χ^2^ = 4.290	0.038
Antihistamine use [yes, n (%)]	21 (25.00)	32 (9.52)	χ^2^ = 14.601	<0.001
Hospital department [internal medicine, n (%)]	35 (41.67)	92 (27.38)	χ^2^ = 6.501	0.011
Hospital department [surgery, n (%)]	25 (29.76)	151 (44.94)	χ^2^ = 6.360	0.011
Hospital department [other, n (%)]	24 (28.57)	93 (27.68)	χ^2^ = 0.026	0.870
Surgery type [emergency, n (%)]	27 (32.14)	66 (19.64)	χ^2^ = 6.091	0.014
Surgery type [elective, n (%)]	28 (33.33)	102 (30.36)	χ^2^ = 0.278	0.598
Surgery type [no surgery, n (%)]	29 (36.90)	168 (50.00)	χ^2^ = 6.463	0.011

## 3 Results

### 3.1 Comparison of baseline characteristics

The baseline characteristics of the observation and control groups are compared in Table 1. The observation group exhibited a significantly higher mean age, a greater prevalence of transfusion history, and an increased number of prior transfusions (all *P* < 0.05). Additionally, the observation group demonstrated significantly higher rates of hypertension, diabetes mellitus, cardiovascular disease, malignancy, and antihistamine use (all *P* < 0.05). Significant differences were also observed in the distribution of hospital departments and surgery types, as detailed in Table 1. These imbalances are expected in an unmatched design and were addressed by including these variables in the subsequent multivariate adjustment. In contrast, no statistically significant differences were found between the groups in terms of gender, body mass index (BMI), the transfusion indications of trauma, anemia, or other, the ‘other’ hospital department category, or elective surgery type (all *P* > 0.05).

### 3.2 Comparison of transfusion history and blood product characteristics

Variables related to specific blood product types (e.g., RBCs, plasma) were analyzed descriptively (Table 2) but were not included in the multivariate patient-level risk factor model due to potential confounding by clinical indication and department. Table 2 presents a comparison of transfusion history and blood product characteristics between the observation and control groups. The observation group demonstrated a significantly higher number of transfusions and transfusion volume, alongside a shorter time since last transfusion, compared to the control group (*P* < 0.05). Regarding blood product types, the observation group had a significantly higher proportion of RBC transfusions but a lower proportion of plasma transfusions (*P* < 0.05). No statistically significant differences were observed between groups for platelets, cryoprecipitate, other blood products, storage duration, or infusion rate (*P* > 0.05) (see Table 2).

**TABLE 2:** Comparison of transfusion history and blood product characteristics.

Variable	Observation group (*n* = 84)	Control group (*n* = 336)	Statistic	*P*-value
Number of transfusions	2.53 ± 1.23	1.82 ± 0.91	*t* = 5.927	<0.001
Time since last transfusion (months)	6.35 ± 4.17	8.87 ± 5.23	*t* = 4.100	<0.001
Blood product type (RBCs, n (%))	36 (42.86)	100 (29.76)	χ^2^ = 5.263	0.022
Blood product type (plasma, n (%))	27 (32.14)	151 (44.94)	χ^2^ = 4.507	0.034
Blood product type (platelets, n (%))	14 (16.67)	54 (16.07)	χ^2^ = 0.017	0.895
Blood product type (cryoprecipitate, n (%))	1 (1.19)	3 (0.89)	Fisher	0.999
Blood product type (other, n (%))	6 (7.14)	18 (5.36)	Fisher	0.683
Transfusion volume (units)	2.83 ± 0.92	2.47 ± 0.83	*t* = 3.478	<0.001
Storage duration (days)	5.24 ± 1.13	5.03 ± 1.02	*t* = 1.650	0.100
Infusion rate (mL/h)	120.47 ± 15.32	118.73 ± 14.79	*t* = 0.958	0.339

### 3.3 Distribution of adverse reaction types

In the observation group, adverse reactions were predominantly allergic and febrile non-hemolytic reactions (see Table 3).

**TABLE 3:** Distribution of adverse reaction types (observation group).

Variable	*n* (%)
Allergic reactions	43 (51.19)
Febrile non-hemolytic reactions	36 (42.86)
Other reactions	5 (5.95)

### 3.4 Comparison of vital signs and laboratory parameters

Table 4 compares the clinical sequelae observed following transfusion, specifically vital signs and laboratory parameters measured at 1 h post-transfusion, highlighting the physiological impact of adverse reactions. The observation group demonstrated significantly higher values in body temperature, heart rate, respiratory rate, systolic blood pressure, white blood cell count, and C-reactive protein compared to the control group (*P* < 0.05). Additionally, the observation group showed significantly lower hemoglobin levels (*P* < 0.05). No statistically significant differences were observed between groups for diastolic blood pressure and platelet count (*P* > 0.05) (see Table 4).

**TABLE 4:** Comparison of vital signs and laboratory parameters (1 h post-transfusion).

Variable	Observation group (*n* = 84)	Control group (*n* = 336)	Statistic	*P*-value
Body temperature (°C)	37.33 ± 0.33	36.87 ± 0.32	*t* = 11.710	<0.001
Heart rate (beats/min)	93.63 ± 10.27	80.35 ± 8.73	*t* = 12.020	<0.001
Respiratory rate (breaths/min)	22.53 ± 3.13	18.87 ± 2.79	*t* = 10.488	<0.001
Systolic blood pressure (mmHg)	130.53 ± 15.23	125.27 ± 14.79	*t* = 2.898	0.004
Diastolic blood pressure (mmHg)	80.33 ± 10.13	78.87 ± 9.82	*t* = 1.211	0.226
Hemoglobin (g/dL)	9.83 ± 1.23	10.17 ± 1.13	*t* = 2.422	0.016
White blood cell count (× 10^9^/L)	8.53 ± 2.13	7.83 ± 1.92	*t* = 2.922	0.004
Platelet count (× 10^9^/L)	150.33 ± 45.63	155.27 ± 42.37	*t* = 0.940	0.347
C-reactive protein (mg/L)	12.53 ± 3.23	8.87 ± 2.83	*t* = 10.296	<0.001

### 3.5 Comparison of clinical management and outcomes

Table 5 details the clinical management strategies employed and the subsequent patient outcomes following the transfusion episode, illustrating the healthcare resource utilization and prognosis associated with adverse reactions. The observation group demonstrated significantly higher rates of antipyretic-analgesic use, corticosteroid use, and ICU monitoring, longer hospital stay, and significantly different outcome distributions with lower complete recovery but higher improvement rates compared to the control group (*P* < 0.05). No statistically significant differences were observed for outcomes of no change and mortality (*P* > 0.05) (see Table 5).

**TABLE 5:** Comparison of clinical management and outcomes.

Variable	Observation group (*n* = 84)	Control group (*n* = 336)	Statistic	*P*-value
Antipyretic-analgesic use [n (%)]	50 (59.52)	17 (5.06)	χ^2^ = 148.384	<0.001
Corticosteroid use [n (%)]	25 (29.76)	7 (2.08)	χ^2^ = 73.142	<0.001
Duration of adverse reactions (hours)	4.53 ± 1.23	–	–	–
ICU monitoring [n (%)]	14 (16.67)	6 (1.79)	Fisher	<0.001
Hospital stay (days)	10.53 ± 3.23	7.83 ± 2.91	*t* = 7.436	<0.001
Outcome [complete recovery, n (%)]	68 (80.95)	312 (92.86)	χ^2^ = 11.054	<0.001
Outcome [improvement, n (%)]	11 (13.10)	13 (3.87)	χ^2^ = 10.624	0.001
Outcome [no change, n (%)]	4 (4.76)	10 (2.98)	–	0.600
Outcome [mortality, n (%)]	1 (1.19)	1 (0.29)	–	0.720

### 3.6 Multivariate logistic regression analysis

**FIGURE 1:**
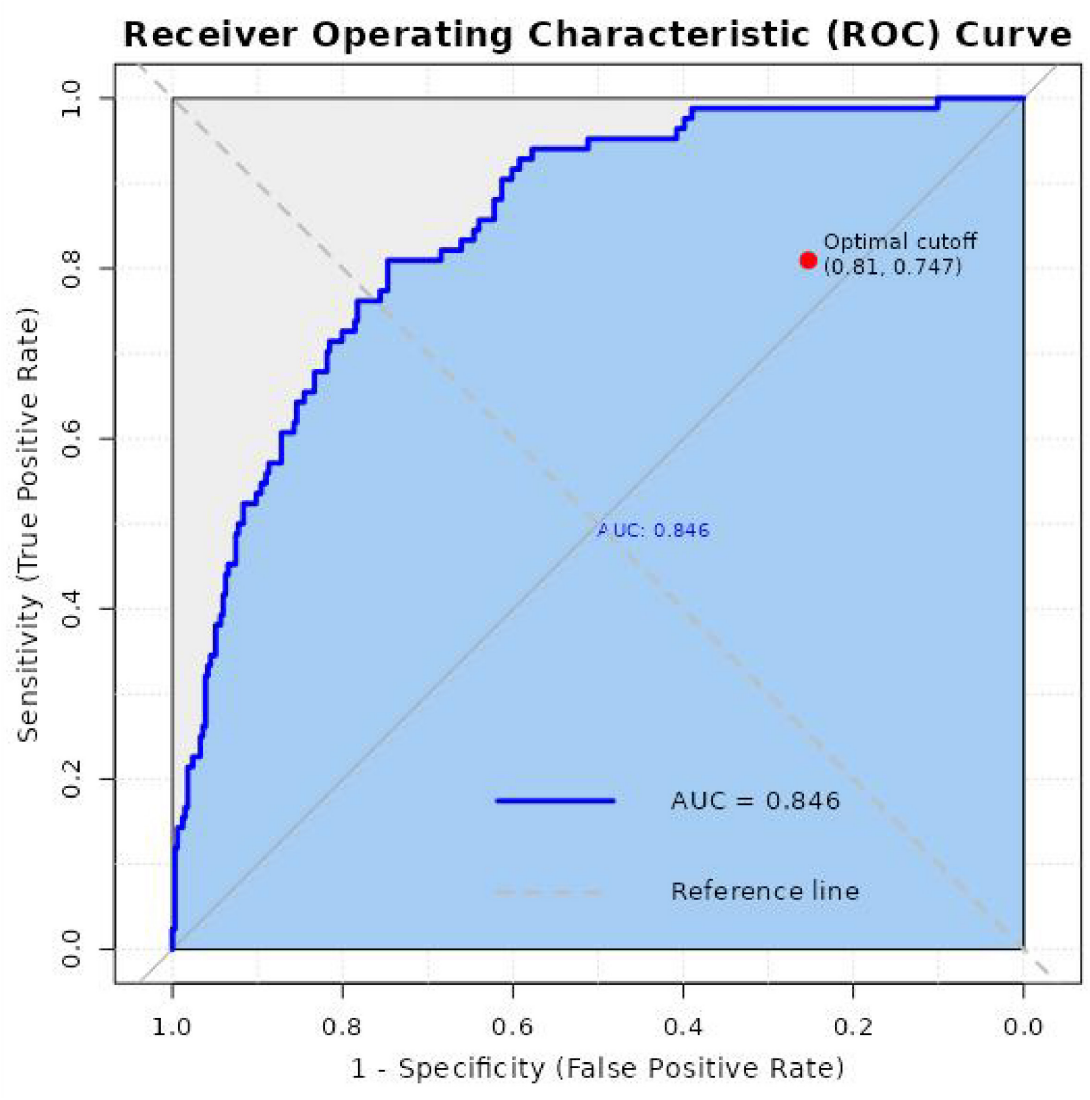
ROC curve demonstrating the discriminatory ability of the nomogram prediction model (AUC = 0.846).

Independent risk factors for transfusion adverse reactions included: age (adjusted OR = 1.05, 95% CI: 1.03–1.07, *P* < 0.001), number of prior transfusions (adjusted OR = 2.47, 95% CI: 1.98–3.08, *P* < 0.001), hypertension (adjusted OR = 1.53, 95% CI: 1.02–2.29, *P* = 0.037), cardiovascular disease (adjusted OR = 2.12, 95% CI: 1.34–3.37, *P* = 0.001), malignancy (adjusted OR = 1.80, 95% CI: 1.04–3.12, *P* = 0.036), and transfusion volume (adjusted OR = 1.65, 95% CI: 1.32–2.05, *P* < 0.001). The Hosmer-Lemeshow test indicated a good fit for the model (χ^2^ = 7.24, *P* = 0.51). The area under the receiver operating characteristic (ROC) curve for this model was 0.846, demonstrating good discriminatory ability. Prior to model refinement, multicollinearity assessment of the initial variables confirmed a high VIF (> 10) between transfusion history and number of prior transfusions. Therefore, only number of prior transfusions was carried forward for the stepwise selection process. The final model results are presented in Table 6 and Figure 1.

**TABLE 6:** Results of multivariate logistic regression.

Characteristic	Adjusted OR	95% CI	*p*-value
Age (per year)	1.05	1.03, 1.07	< 0.001
Number of prior transfusions (per unit)	2.47	1.98, 3.08	< 0.001
Hypertension (Yes vs. No)	1.53	1.02, 2.29	0.037
Diabetes mellitus (Yes vs. No)	1.62	1.01, 2.60	0.045
Cardiovascular disease (Yes vs. No)	2.12	1.34, 3.37	0.001
Malignancy (Yes vs. No)	1.80	1.04, 3.12	0.036
**Hospital department (Ref: Other)**			
- Internal Medicine	2.01	1.18, 3.43	0.01
Surgery	1.15	0.66, 2.00	0.624
Transfusion volume (per unit)	1.65	1.32, 2.05	< 0.001

### 3.7 Subgroup analyses

To explore risk patterns in specific populations, subgroup analyses were performed. In patients receiving Red Blood Cells (RBCs, *n* = 146), significant risk factors included transfusion history (OR = 9.45, *P* < 0.001) and transfusion volume (OR = 1.58, *P* = 0.008). In patients receiving Plasma (*n* = 178), age (OR = 1.06, *P* = 0.002) and cardiovascular disease (OR = 2.89, *P* = 0.003) were significant. Stratifying by transfusion history, in patients with a prior history (*n* = 208), the number of previous transfusions remained a strong predictor (OR = 2.55, *P* < 0.001), while in those without a prior history (*n* = 212), malignancy emerged as a significant risk factor (OR = 2.65, *P* = 0.047).

## 4 Discussion

This study leveraged 5-year real-world data from a tertiary hospital to systematically examine factors influencing adverse transfusion reactions, with a primary focus on identifying high-risk populations and refining transfusion safety protocols. The results demonstrated a low overall incidence of adverse transfusion reactions; however, multivariate analysis revealed that age, transfusion history, type of blood product administered, and transfusion volume served as independent risk factors. These findings underscore the necessity of integrating patient baseline characteristics and transfusion specifics into clinical decision-making to mitigate reaction risks. Consistent with prior investigations, this work affirms that real-world data can effectively elucidate the multifactorial etiology of transfusion reactions, thereby informing personalized transfusion strategies (16).

In comparative analyses of baseline characteristics, patients in the observation group exhibited advanced age, more frequent transfusion histories, and a higher burden of comorbidities, suggesting that elderly individuals and those with repeated transfusions may be predisposed to reactions due to immunological alterations or sensitization effects (17, 18). Additionally, imbalances in prophylactic antihistamine usage were observed. It is crucial to interpret this finding with caution, as pre-transfusion antihistamine administration typically reflects a clinician’s assessment of high baseline risk (e.g., prior allergic reaction) rather than being an independent causative factor. This highlights a potential confounding by indication, which was addressed by excluding this variable from the final predictive model.

Analyses of transfusion history and blood product attributes revealed that the observation group had a greater number of prior transfusions, shorter intervals since the last transfusion, and higher proportions of red blood cell and plasma transfusions, implying that recurrent transfusions may foster immune memory through antigen exposure, elevating the likelihood of allergic or febrile reactions (19, 20). Although no significant differences were observed in blood product storage duration or infusion rates, higher transfusion volumes correlated with adverse reactions, indicating that cumulative exposure might directly activate humoral or cellular immune responses (21). These outcomes align with AABB guidelines, which emphasize limiting non-essential transfusions and prioritizing low-sensitization blood products in transfusion planning (22).

The distribution of adverse reaction types was predominantly characterized by allergic and febrile reactions, potentially attributable to residual leukocytes, platelets, or plasma proteins in blood products triggering histamine release or cytokine storms (23). Hemolytic reactions occurred infrequently; nevertheless, vigilance against acute hemolysis risks due to ABO incompatibility or storage-related damage remains crucial (24). Other reactions, such as circulatory overload, though rare, carried severe consequences in elderly or cardiocompromised patients, underscoring the imperative for intensified vital sign monitoring during transfusions (25). This pattern corroborates that transfusion reactions often stem from immune-mediated mechanisms rather than singular causative factors.

Vital signs and laboratory parameters assessed 1 h post-transfusion revealed significantly elevated body temperature, heart rate, respiratory rate, as well as C-reactive protein and total serum IgE levels in the observation group, underscoring the pivotal role of acute inflammatory and allergic responses in the underlying pathophysiology (26). Minor alterations in hemoglobin and leukocyte counts potentially reflect hemodilution or stress responses, whereas the absence of significant change in platelet count suggests a more frequent involvement of humoral rather than cellular components in these reactions (27). These parameters may serve as early warning indicators, facilitating timely clinical intervention to mitigate reaction progression (28).

Comparative analysis of clinical management and patient outcomes indicated more frequent administration of antihistamines, antipyretics/analgesics, and corticosteroids in the observation group, alongside prolonged hospitalization and higher ICU admission rates. These findings highlight the substantial negative impact of adverse reactions on healthcare resource utilization and patient prognosis (29). Although the majority of patients achieved full recovery, a subset exhibited delayed improvement or mortality, emphasizing the necessity for optimized reaction management protocols incorporating rapid identification and stepwise therapeutic strategies (30). Outcome disparities were associated with the severity of underlying comorbidities, suggesting that effective comorbidity management constitutes a critical component for enhancing transfusion safety (31).

Multivariable logistic regression analysis identified advanced age, prior transfusion history, plasma transfusion, and transfusion volume as independent risk factors. Among these, transfusion history demonstrated the highest odds ratio, highlighting the significance of previous antigen exposure in the sensitization process. The elevated risk associated with plasma transfusion may be attributable to its higher content of foreign proteins, while increased transfusion volume likely amplifies immune activation through a dose-response relationship (32). The model exhibited good fit, providing an empirical foundation for developing risk assessment tools, though validation in larger, diverse cohorts is warranted to enhance generalizability. The most potent risk factor identified was a history of prior transfusion (OR = 11.21), a finding with strong biological plausibility. Repeated exposure to allogeneic blood products can drive alloimmunization, leading to the formation of antibodies against foreign antigens on red blood cells (e.g., in the Rh, Kell, Kidd systems), leukocytes, platelets, or plasma proteins. For febrile non-hemolytic reactions (FNHTR), prior sensitization to donor leukocytes is a key mechanism; subsequent transfusions containing leukocytes or leukocyte-derived cytokines can trigger an anamnestic response, resulting in pyrogen release. For allergic reactions, prior exposure may induce IgE antibodies against soluble proteins in donor plasma (e.g., IgA, haptoglobin). Upon re-exposure, antigen cross-linking of IgE on mast cells and basophils leads to rapid histamine release. This study’s finding that the number of prior transfusions was also an independent risk factor (OR = 2.47) supports a dose-response relationship consistent with cumulative sensitization. Furthermore, the protective effect of a longer time since last transfusion (OR = 0.85) might reflect the waning of transient, low-titer antibodies or immune tolerance mechanisms over time, though this requires further investigation. These mechanisms underscore the critical importance of obtaining a detailed transfusion history and considering strategies such as leukoreduction, prophylactic medication (with the caveats noted earlier), or, where feasible, the use of antigen-matched products for patients with prior transfusion exposure.

### 4.1 Study limitations

This study has several limitations inherent to its design and data source. Firstly, as a retrospective analysis of single-center RWD, it is susceptible to information biases such as under-reporting of mild adverse reactions, inconsistencies in documentation, and missing data for some potential confounders (e.g., detailed genetic or immunological profiles). Secondly, the unmatched case-control design, while efficient, resulted in baseline imbalances between groups. Although we adjusted for these in multivariate analysis, residual confounding cannot be ruled out. The single-center setting limits the generalizability (external validity) of our findings to other hospitals with different patient demographics, transfusion practices, and hemovigilance reporting standards. Thirdly, the measurement of antihistamine use is a clear example of confounding by indication, limiting its causal interpretation. Lastly, the cross-sectional nature of the analysis precludes establishing definitive temporal causality. Future prospective, multi-center studies with standardized data collection protocols are needed to validate these risk factors and develop broadly applicable prediction models.

## 5 Conclusion

In summary, this real-world investigation elucidates that transfusion-associated adverse reactions are driven by multiple factors, including patient age, transfusion history, comorbid conditions, and blood product characteristics. Clinical practice should emphasize enhanced screening of high-risk populations and the implementation of individualized transfusion strategies. These findings not only align with current transfusion safety guidelines but also provide direction for refining the prevention and management of adverse reactions, ultimately contributing to improved patient safety and healthcare quality.

## Data Availability

The original contributions presented in the study are included in the article/supplementary material, further inquiries can be directed to the corresponding authors.
